# The Role of Diabetes in Acromegaly Associated Neoplasia

**DOI:** 10.1371/journal.pone.0127276

**Published:** 2015-05-21

**Authors:** Sonia Cheng, Karen Gomez, Omar Serri, Constance Chik, Shereen Ezzat

**Affiliations:** 1 Department of Medicine, Division of Endocrinology and Metabolism, University of Alberta, Edmonton, Alberta, Canada; 2 Department of Medicine, University of Toronto, Toronto, Ontario, Canada; 3 Department of Medicine, University of Montreal, Montréal, Québec, Canada; University of Cordoba, SPAIN

## Abstract

**Background:**

The risk and mortality due to cancer in patients with acromegaly have been previously investigated. Although GH/IGF-1 excess provides a probable pathophysiological explanation, the degree of IGF-1 excess and the role in acromegaly-associated neoplasms of diabetes, a common comorbidity in acromegaly with known association with cancer, remains unclear.

**Methods:**

Acromegalic patients treated in three Canadian referral centers (Toronto, Montreal, Edmonton) were included. All available clinical information was recorded including: age, initial and last percentage of the upper limit of normal (%ULN) IGF-1 levels, comorbidities and other neoplasms (benign and malignant).

**Results:**

408 cases were assessed. 185 were women (45.3%), 126 (30.9%) developed extra-pituitary neoplasms: 55 malignant and 71 benign. The most frequent anatomic site was the gastrointestinal tract (46 [11.3%]), followed by head and neck (36 [8.8%]) and multiple locations (14 [3.4%]). 106 (26.0%) cases had diabetes. Initial IGF-1 was significantly higher in men older than 50 (380.15 vs. 284.78, p = 0.001) when compared to men younger than 50. Diabetics showed significantly higher initial IGF-1 (389.38 vs. 285.27, p = 0.009), as did diabetics older than 50 compared with those without diabetes. 45.3% (48/106) of cases with diabetes developed extra-pituitary neoplasms vs. 24.3% (71/292) without diabetes (p = 0.001, OR: 2.576 95%CI 1.615–4.108). 22.6% (24/106) of cases with diabetes developed malignant tumors vs. 9.2% (27/292), (p < 0.001, OR 2.873, 95%CI 1.572–5.250).

**Conclusions:**

These data suggest that acromegalic patients with diabetes are more likely to develop extra-pituitary neoplasms and their initial IGF-1 levels are higher. The contribution of IGF-1 vs. diabetes alone or in combination in the development of extra-pituitary neoplasms warrants further investigation.

## Introduction

Acromegaly is a disease characterized by excessive growth hormone (GH) most frequently due to a GH-secreting pituitary adenoma [1,2]. Multiple clinical observations have suggested the association of acromegaly and cancer development, both in risk and mortality [3,4].

Chronic stimulation by GH/insulin-like growth factor-1 (IGF-1) has been described as a potential pathophysiologic explanation [5], with clinical evidence available outside of the acromegaly setting, for breast cancer [6], colorectal cancer [7] and prostate cancer [8]. In some cases, IGF-1 levels have been associated with disease progression [7].

In patients with acromegaly, although clinical observations have been debated mainly due to epidemiological confounders [9,10], series reporting on the association between acromegaly and malignancy remain controversial [4]. Nevertheless, acromegaly has been linked to an increased risk of thyroid cancer, colonic polyps and urinary tract carcinomas [3,11,12].

Animal models support the participation of the GH/IGF-1 axis in the development of neoplasms, both in tumor growth and metastases [13]. IGF-1 mainly affects cell-cycle progression through the phosphatidylinositol 3-kinase (PI-3K)/protein kinase B (AKT) and extracellular signal-regulated kinases (ERK) pathways [14]. Human studies have also identified increased genomic instability in association with IGF-1 levels in patients with acromegaly, suggesting an alternative oncogenic mechanism [15].

Impaired glucose tolerance and diabetes are among the most frequent acromegaly comorbidities [16]. IGF-1 levels have been associated with insulin resistance and hyperglycemia [17]. Our group has previously described the association of diabetes with pituitary pathology and therapeutic outcomes in patients with acromegaly [18].

In this study we examined the association of diabetes and cancer risk in patients with acromegaly.

## Patients and Methods

We included all consecutive patients treated for acromegaly from three Canadian referral centers: University Health Network, Toronto; Centre Hospitalier de l’Université de Montréal, Montreal; and University of Alberta Hospital, Edmonton. Cases were identified in each hospital from dedicated neuroendocrinology clinics receiving referrals from large areas of each province; each patient was systematically followed for surveillance of symptoms, biochemical disease control and presence of acromegaly comorbidities including other malignancies.

We used defined prevailing screening protocols to assess all comorbidities. There was no difference in the routine screening for acromegalic patients with or without diabetes in the referral centers.

Patients were designated as having diabetes only when persistent hyperglycemia was present after initial acromegaly treatment, i.e. biochemical remission or improvement was obtained. This was necessary to distinguish them from those where the diabetes resolved with GH levels improvement.

With approval of each institution’s review board (Institutional Review Board, University Health Network, 11-0489-CE; Comité d'éthique de la recherche du CHUM, SL 06.098—BSP and University of Alberta, Research Ethics Office Pro00042711), all information was anonymized and de-identified. No informed consent was required. We recorded clinical information from all available medical records, including: age at diagnosis, gender, initial and final IGF-1 levels, co-morbidities and history of other neoplasms (benign and malignant). IGF-1 is presented as the percentage of the upper limit of normal for age and gender (%ULN).

Statistical analysis was performed with SPSS v21 software. For proportion comparisons we used chi-square and risk tests, and Fisher’s test when applicable. For comparisons of means we used Student’s T or Mann-Whitney U according to the analyzed variables distribution. Continuous variables are presented as means; their ranges are shown in brackets. Statistical significance was considered with a p-value below 0.05. All comparisons with a p-value above 0.05 are shown as non-significant (NS).

## Results

We identified 408 cases from the three referral centers, 184 cases from Toronto, 107 from Montreal, and 117 from Edmonton. 185 were women (45.3%) and 223 men (54.7%). Mean age at diagnosis of acromegaly was 43.2 (13–79). 170 were younger than 50 (41.7%). Mean duration of follow up was 10.2 years (0–43).

Of these, 126 (30.9%) developed extra-pituitary neoplasms, 55 malignant and 71 benign. The most frequent anatomic site for overall tumors was the gastrointestinal tract (47 [11.5%]), followed by head and neck (36 [8.8%]) and multiple locations (14 [3.4%]) (i.e. cases with more than one primary location of extra-pituitary tumors). Benign tumors appeared more frequently in the gastrointestinal tract (44/71, 62.0%) followed by head and neck growths (23/71, 32.4%). Malignant neoplasms were more frequent in the head and neck (13/55, 23.6%) and the genitourinary tract (13/55, 23.6%) followed by multiple locations (12/55, 21.8%), [Table 1]. Thyroid cancers were included in the Head and Neck cancers. Thyroid nodules, including multi-nodular goiters, were considered benign unless pathology demonstrated malignancy.

**Table 1:** Location of extra-pituitary tumors and distribution according to malignancy.

Location	N	%	Malignant N (%)	Benign N (%)
No tumor	282	69.11	-	-
Head and neck	36	8.82	13 (23.63)	23 (32.39)
GI tract	47	11.51	3 (5.45)	44 (61.97)
GU tract	14	3.43	13 (23.63)	1 (1.4)
Multiple locations	14	3.43	12 (21.81)	2 (2.81)
Skin	6	1.47	5 (9.09)	1 (1.4)
Hematologic	5	1.22	5 (9.09)	0
Chest	4	0.98	4 (7.27)	0
Total	408	100	55 (100)	71 (100)

GI: gastrointestinal, GU: genitourinary.

In cases with complete information, we identified that: 20/25 cancer cases occurred simultaneously or after the diagnosis of acromegaly, among these cases, 13 were diagnosed following acromegaly remission. Four cases were identified with 2 cancer diagnoses and one with 3 cancers. Five cancers were identified before the diagnosis of acromegaly. Eleven cases had the diagnosis of acromegaly and cancer within 5 years, 5 from 5 to 10 years, 7 from 10 to 20 years and 2 after more than 20 years of the diagnosis of acromegaly.

### Extra pituitary neoplasms and gender

From the 408 cases, 53/185 (28.6%) women and 73/223 (32.7%) men developed extra pituitary neoplasms (NS). Benign tumors appeared in 29/185 (15.7%) women and in 42/223 (18.8%) men (NS). Malignant tumors were described in 24/185 (13.0%) women and 31/223 (13.9%) men (NS).

### Extra pituitary neoplasms and age groups

From 406 cases with available information, 39/170 (22.9%) below the age of 50 developed extra-pituitary tumors compared with 86/236 (36.4%) above 50 years of age (p = 0.002). Benign tumors were found in 26/170 (15.3%) below 50 vs. 45/236 (19.1%) in the older group (NS). Malignant tumors were described in 13/170 (7.6%) younger cases as opposed to 41/236 (17.4%) in the older age group (p = 0.003).

### Extra pituitary neoplasms by gender and age group

In women with acromegaly, 15/70 (21.4%) cases below age 50 developed other tumors as opposed to 38/114 (33.3%) in the older age group (p = 0.058). In the group of men, 24/100 (24%) younger cases developed extra pituitary tumors as opposed to 48/122 (39.3%, p = 0.011) in the older group. Benign tumors occurred in 11/70 (15.7%) of younger women and 18/114 (15.8%) of older women (NS). They were described in 15/100 (15.0%) of younger men as opposed to 27/122 (22.1%) of older men (NS). Malignant tumors were described in 4/70 (5.7%) younger women as opposed to 20/114 (17.5%) in older women (p = 0.015). They were present in 9/100 (9%) younger men vs. 21/122 (17.2%) older men (p = 0.055).

### Diabetes, gender and age group

From cases with available information, 107/398 (26.0%) had the diagnosis of diabetes, 43/181 women (23.8%) had diabetes as opposed to 63/217 men (29.0%; NS).

From the cases under 50 years of age, 28/164 (17.1%) had the diagnosis of diabetes vs. 78/233 from those older than 50 (33.5%; p < 0.001).

In the group of women, 10/68 (14.7%) cases younger than 50 had the diagnosis of diabetes as opposed to 33/112 (29.5%) of women older than 50 (p = 0.018). In the group of men, 18/96 cases younger than 50 (18.8%) had diabetes vs. 45/121 of those older than 50 (37.2%; p = 0.02).

Pharmacological treatment of diabetes was available in 16/25 patients. Analyzed in one of the cohorts, (N = 30) HbA1c was 8.24% (SD = 4.0) for cases with malignant tumors, 6.3% (SD = 0.91) for cases with benign tumors and 7.1% (SD = 2.3) for cases without neoplasms. Mean HbA1c after acromegaly treatment (N = 38) was 6.3% (SD = 0.96) for cases with malignant tumors, 6.4% (SD = 0.81) for cases with benign tumors and 6.5% (SD = 1.0) for cases without neoplasms. While these values are of suggestive of a trend, particularly for a difference between benign and malignant lesions before acromegaly treatment, this did not reach statistical significance.

### Extra-pituitary neoplasms and diabetes

Acromegalic patients with diabetes developed extra-pituitary tumors more frequently: 48/106 (45.3%) as opposed to 71/292 (24.3%) in cases without diabetes (p < 0.001; OR = 2.576, 95%CI 1.615–4.108), [Table 2, middle panel, left]. Likewise, diabetic patients showed a higher frequency 24/106 (22.6%) of malignant tumors, compared to 27/192 (9.2%) those without diabetes (p = 0.001; OR = 2.873, 95%CI 1,572–5.250). Benign tumors showed a similar trend without reaching statistical significance (24/106, 22.6% vs. 44/292, 15.1%, p = 0.054). Overall tumors were significantly more frequent in diabetic cases for both age groups. Malignant tumors were significantly more frequent for the older age group [Table 2]. Multinomial logistic regression revealed both age (p = 0.009 for age 50 or below) and diabetes (p = 0.008) as significantly associated with the presence of overall tumors and with malignant tumors (p = 0.005 for diabetes and p = 0.025 for age 50 or below).

**Table 2:** Extra-pituitary and malignant tumors according to age, diabetes and both.

		Extra-pituitary tumor		Malignant tumor	
		(%)*	p	(%)*	p
Age	< 50	22.9	0.002	7.64	0.003
	> 50	36.44		17.37	
Diabetes	Yes	45.28	<0.001	22.64	0.001
	No	24.31		9.24	
Age + Diabetes	< 50 DM	35.71	0.050	10.71	NS
	< 50 No DM	19.11		6.61	
	> 50 DM	48.71	0.003	26.58	0.003
	> 50 No DM	29.03		11.68	

< 50: younger than 50 years of age; > 50: 50 years or older. DM: type 2 diabetes.

* Percentages shown represent percentage within each compared group.

No significant difference was found in the frequency of extra-pituitary tumors when considering the different diabetes treatments (metformin vs. insulin) received.

### IGF-1

**Fig 1:**
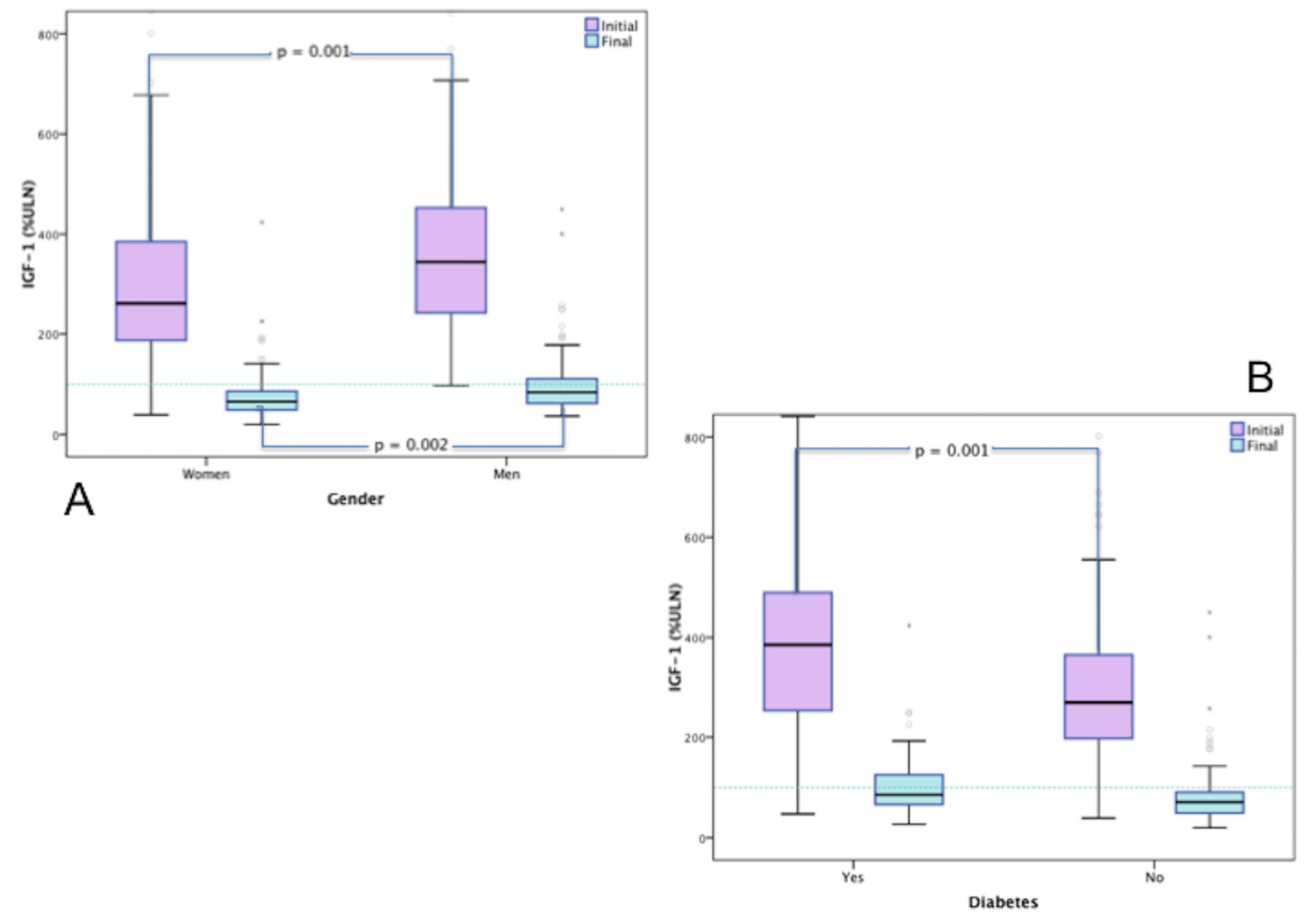
Initial and final IGF-1 (%ULN). **A.** Initial IGF-1 was significantly higher in men (343.82 vs. 277.00, p = 0.001) as was Final IGF-1 (100.77 vs. 81.26, p = 0.022). **B.** Initial IGF-1 was significantly higher in cases with diabetes (378.50 vs. 284.41, p = 0.001).

Both initial (343.8 vs. 277.0, p = 0.001) and final %ULN IGF-1 (100.7 vs. 81.2, p = 0.022) were significantly higher for men when compared to women [Fig 1A].

Initial IGF-1 showed no significant difference for cases with and without malignant tumors. It showed no significant difference for cases with and without extra-pituitary tumors between genders (Women: 282.46 vs. 276.18 and Men: 337.64 vs. 344.86, NS).

No significant difference was found in initial IGF-1 for extra-pituitary, benign or malignant tumors according to age groups.

Men older than 50 years displayed a significantly higher initial IGF-1 (380.15 vs. 284.78, p = 0.001) when compared to their younger (< 50) counterparts.

Initial IGF-1 was significantly higher in diabetics (389.38 vs. 285.27, p = 0.001) [Fig 1B] and specifically in older diabetic men (420.19 vs. 329.44, p = 0.017) compared with those without diabetes.

### Cancer risk in acromegaly vs. general population

**Fig 2:**
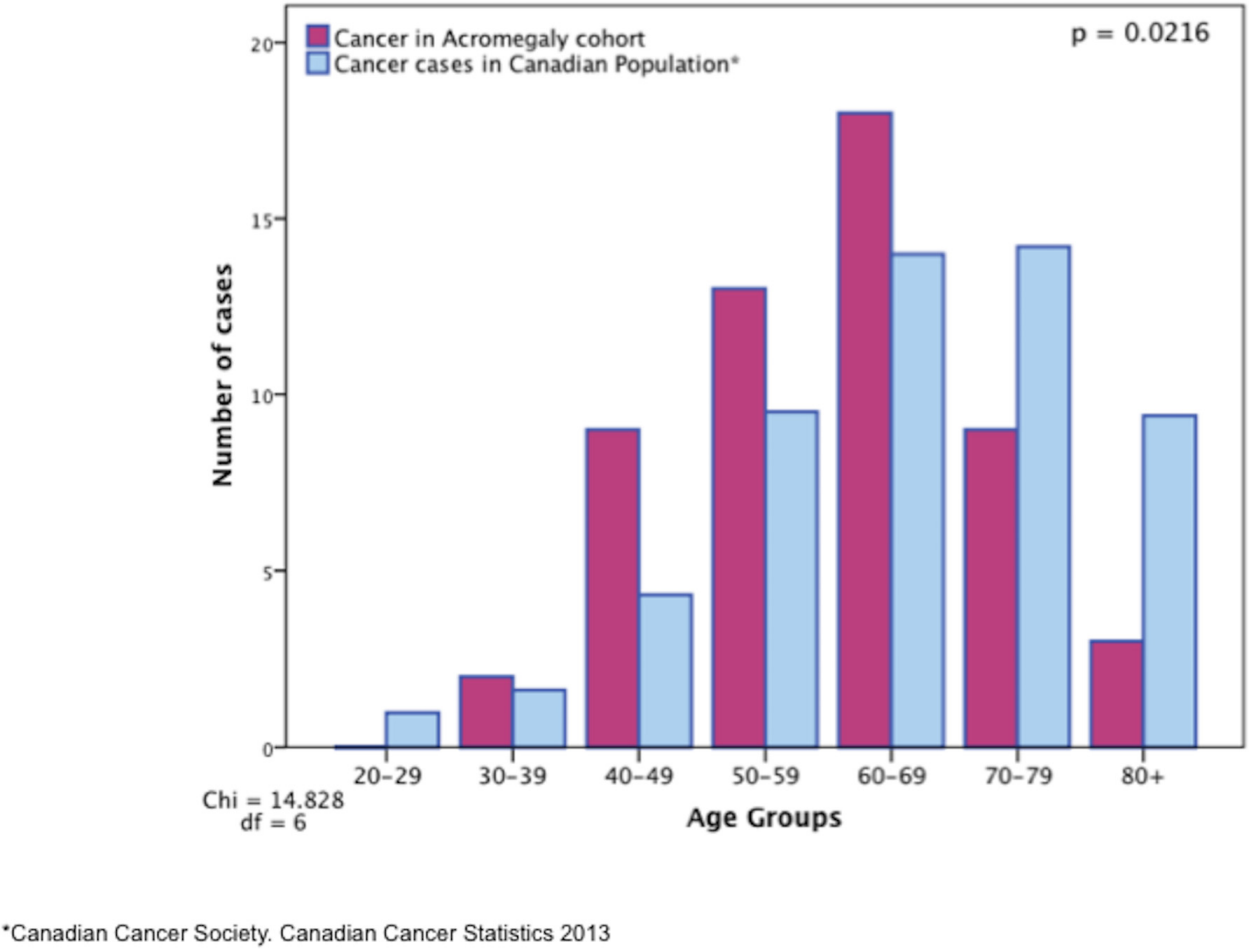
Number of malignant tumors for the study group vs. the general Canadian population according to age groups. **A.** Patients with acromegaly in the younger decades show a significantly higher frequency of cancers when compared to the general population, in the younger age groups.

When compared to Cancer Care reports for the Canadian population [19], the proportions of malignant tumors found for each age group in our cohort are higher for the younger cases than those reported for the general population as ten-year tumor based prevalence. Acromegalics younger than 50 years show a higher proportion of malignant tumors than expected according to the general population’s distribution. Interestingly, and for unclear reasons, the opposite trend appears for cases above 50 years of age [Fig 2].

## Discussion

In this multicenter study we evaluated the association between acromegaly, diabetes, and cancer. Our results show as expected, that acromegalic patients have a higher prevalence of diabetes than the general population (26% vs. 5.8–12.4%) [20,21]. Additionally, acromegalics with diabetes developed extra-pituitary tumors twice as frequently as non-diabetics. Moreover, diabetic acromegalics developed malignant tumors almost three times as frequently as those without diabetes. Finally, we found that older acromegalics with diabetes have an increased risk of extra-pituitary and malignant tumors.

The association of cancer and diabetes has been previously described in the general population; a causal association could be attributed to the effect of hyperinsulinemia, increased IGF-1, and inflammatory mediators [22]. The role of certain diabetes medications in cancer risk has been suggested but not well defined [23,24]. According to multiple meta-analyses in the general population, the anatomic sites most frequently associated with diabetes are pancreatic cancer (OR 1.8; 95%CI 1.7–1.9), colorectal cancer (RR 1.3; 95%CI 1.20–1.40), breast cancer (RR 1.20; 95%CI 1.12–1.28), endometrial cancer (RR 2.10, 95%CI 1.75–2.53), and bladder cancer (RR 1.24; 95%CI 1.08–1.42) [25]. Others have reported liver cancer (RR 2.50, 95%CI 1.8–3.5) and kidney cancer (RR 2.22, 95%CI 1.04–4.70) as well [26]. It should be noted that the odds ratio for malignant tumors found in our acromegaly cohort (OR = 2.873, 95%CI 1.572–5.250) is higher than those previously reported for diabetes alone [25]. This suggests that acromegaly likely potentiates the risk of cancer in patients with diabetes.

As possible mechanisms, animal models have identified the mitogenic and transforming role of different elements of the GH/IGF-1 axis [13,26]. The IGF-1 receptor (IGF-1R) plays a crucial role by activating several intracellular signaling cascades including the Insulin receptor substrate-1 (IRS-1), Src homology domain containing (Shc) and the receptor for activated C kinase 1 (RACK-1). Their activation triggers a nuclear transcriptional response prompting cellular growth, proliferation, motility and invasion [13]. Furthermore, high glucose has been shown to alter signaling pathways of IGF-1 by reducing its metabolic effect and enhancing its proliferative pathway [27]. The close homology of the IGF-1 receptor to the insulin receptor is relevant in this context since both share the same downstream effectors. Whether diabetes increases cancer risk through hyperinsulinemia alone or due to its combined effect on insulin and IGF-1 is still unclear. Moreover, therapeutic agents used in diabetes management have been questioned in increasing cancer risk [24]. Conversely, other studies have shown a potential survival advantage for diabetic patients treated with metformin [23].

We should note that we used the IGF-1 age-adjusted %ULN for analysis. Therefore, absolute values of IGF-1 might show similar or even lower levels in older men given the age-related decrease in serum concentrations.

Initial IGF-1 was significantly higher for the group of patients diagnosed with diabetes, as previously shown by our group [28] especially if they were over the age of 50. Patients with diabetes also showed a significantly higher initial IGF-1 level, independent of therapy achieved targets. This would suggest an effect of previous exposure to elevated levels of IGF-1 as well as persistent exposure during treatment in the development of insulin resistance and diabetes. Of note, the relationship between IGF-1 and tumor development was not observed for patients who developed malignant neoplasms. These latter findings suggest an alternate driving force for malignant transformation in acromegalic patients.

The main limitations of our study are its retrospective nature, the limited duration of follow-up for the study of carcinogenesis risk factors and the different screening rates of neoplasms between institutions. As well, a consistent documentation of diabetes control and management is mandatory to refine our conclusions. A retrospective model is required mainly due to the low incidence of acromegaly as this allows for a large range of follow-up times. Studying these cases for a longer period of follow-up would certainly increase the frequency of malignancies. On the other hand, screening colonoscopies and thyroid ultrasounds are not yet performed at a 100% rate at the moment of acromegaly diagnosis in our institutions. The differences in screening rates could account for varying frequencies of the benign conditions described.
